# Supplementary material for: Hazel Leaf Polyphenol Extract Alleviated Cisplatin-Induced Acute Kidney Injury by Reducing Ferroptosis through Inhibiting Hippo Signaling
Source: Molecules. 2024 Apr 11;29(8):1729. doi: 10.3390/molecules29081729 (PMC11051766; doi:10.3390/molecules29081729)
Supplement: Supplementary file 1 [file molecules-29-01729-s001.zip › molecules-2864925-supplementary.pdf]

Table S1. Ingredients contained in hazel leaf polyphenols

Compound	Chemical formula	Structural formula	Classification of compounds	Relative content(×10^4)
Gallic acid	$C_7H_6O_5$	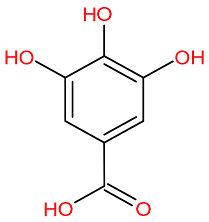	FP, BCP, ACP, BBP, ABP	31.3±6.22
Gallocatechin	$C_{15}H_{14}O_7$	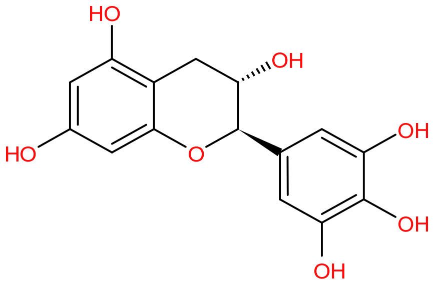	FP	2.79±0.57
2,5-dihydroxybenzoic acid	$C_7H_6O_4$	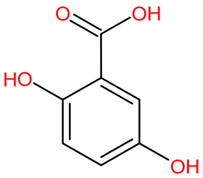	FP, BCP, ABP	5.78±1.26
Chlorogenic acid	$C_{16}H_{18}O_9$	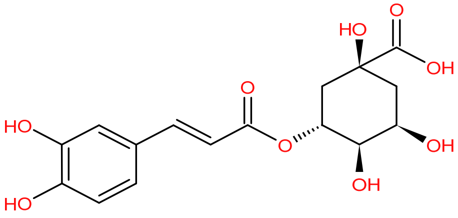	FP	4.13 ± 0.21

Quercetin-3-O-beta-D-glucopyranoside	$C_{21}H_{20}O_{12}$	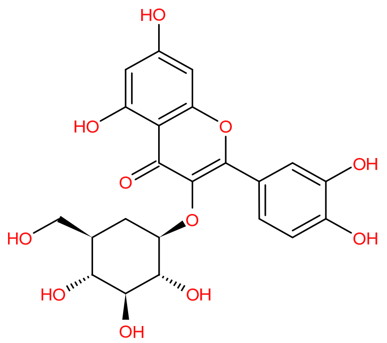	FP, BBP	36.75±7.61
P-Coumaric acid	$C_9H_8O_3$	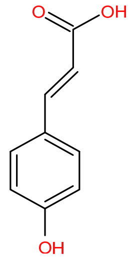	FP, ACP, BBP	16.33±3.89
Myricetin	$C_{15}H_{10}O_8$	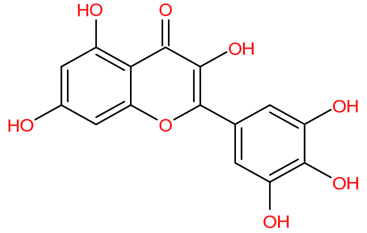	FP, ACP, ABP	74.32±12.87
Pedalitin	$C_{16}H_{12}O_7$	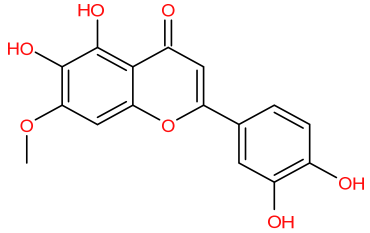	FP	4.23 ± 0.91

Quercetin	$C_{15}H_{10}O_7$	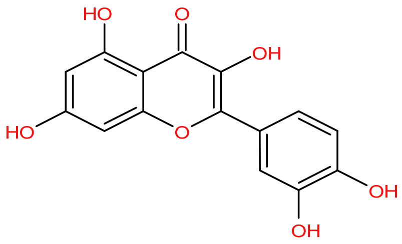	FP, ACP, ABP	154.91±24.28
Luteolin-7-O-glucoside	$C_{21}H_{20}O_{11}$	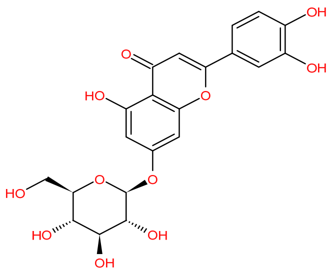	FP, BBP	73.07±18.04
Caffeic acid	$C_9H_8O_4$	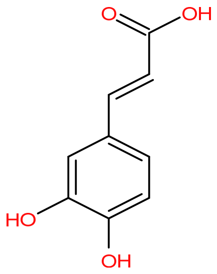	BCP, BBP	14.27±2.07
Resveratrol	$C_{14}H_{12}O_3$	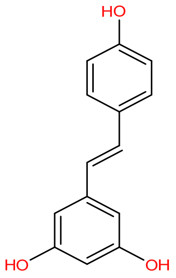	BCP	4.59 ± 0.52
Luteolin	$C_{15}H_{10}O_6$	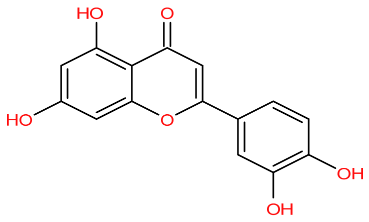	ACP, ABP	21.29±6.22

Ellagic acid	$C_{14}H_6O_8$	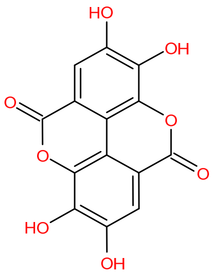	ACP	15.79 ± 4.37
Hydroxybenzoic acid	$C_7H_6O_3$	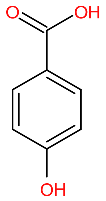	BBP, ABP	6.25±1.30
Quercetin-3-O-beta-D-glucuronide	$C_{21}H_{18}O_{13}$	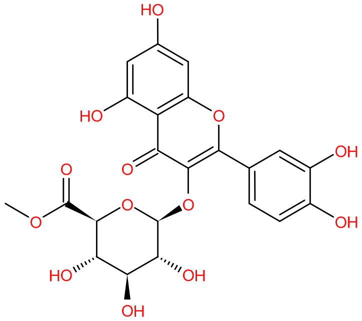	BBP	6.43 ± 0.60
Kaempferol-3-O-rhamnoside	$C_{21}H_{20}O_{10}$	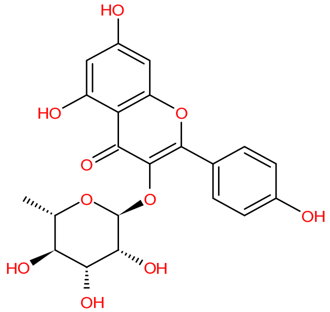	BBP	27.77± 5.36

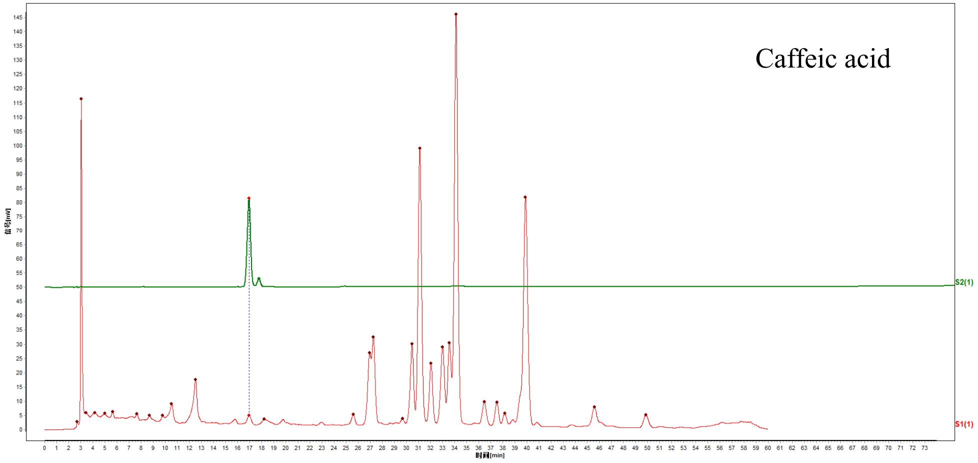
Caffeic acid
S2(1)
S1(1)

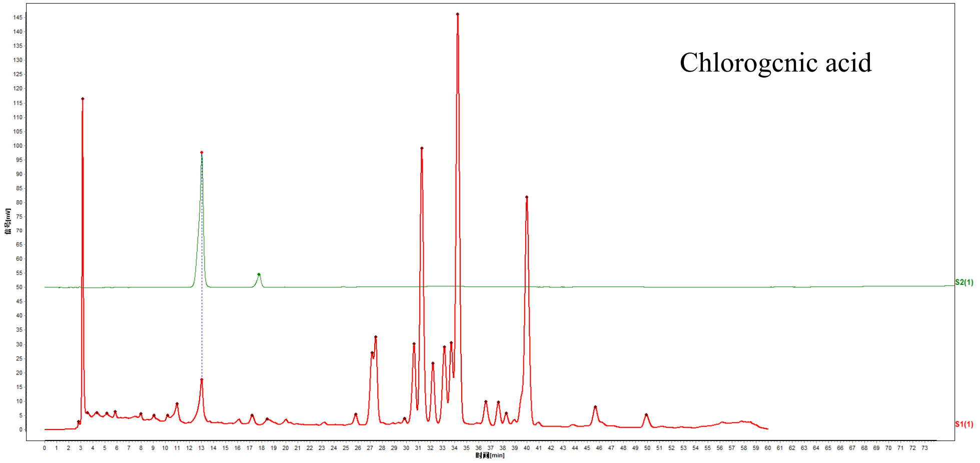
Chlorogcnic acid
S2(1)
S1(1)

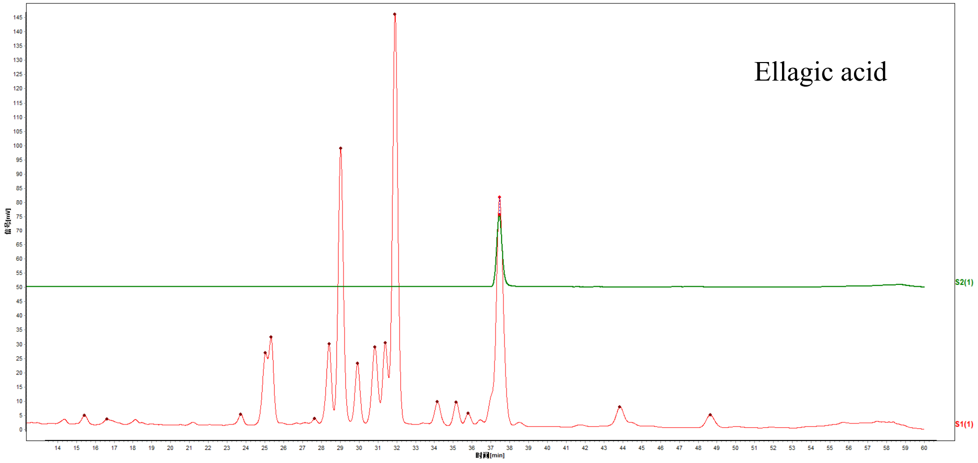
Ellagic acid
S2(1)
S1(1)

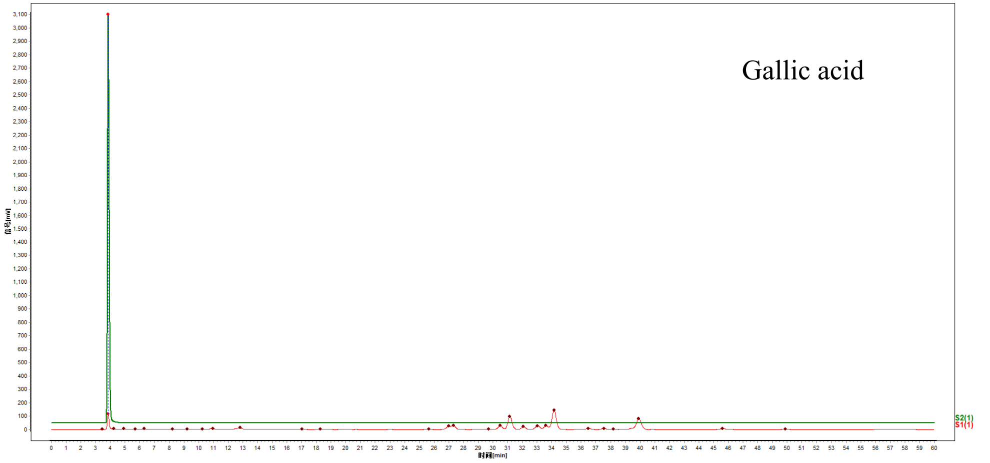
Gallic acid
S2(1)
S1(1)

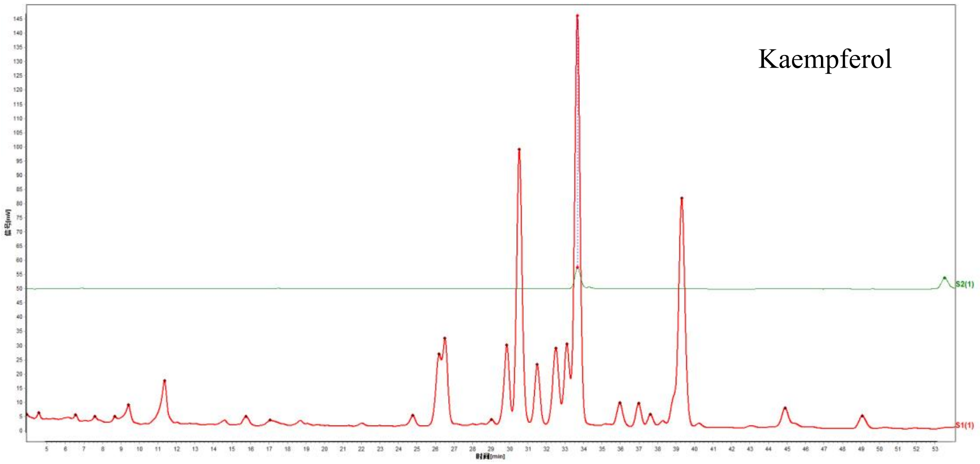
Kaempferol
S2(1)
S1(1)

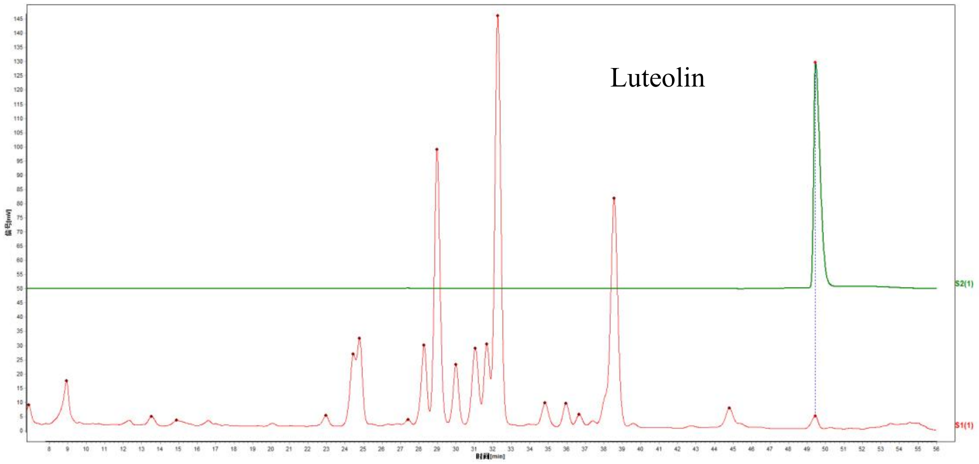
Luteolin
S2(1)
S1(1)

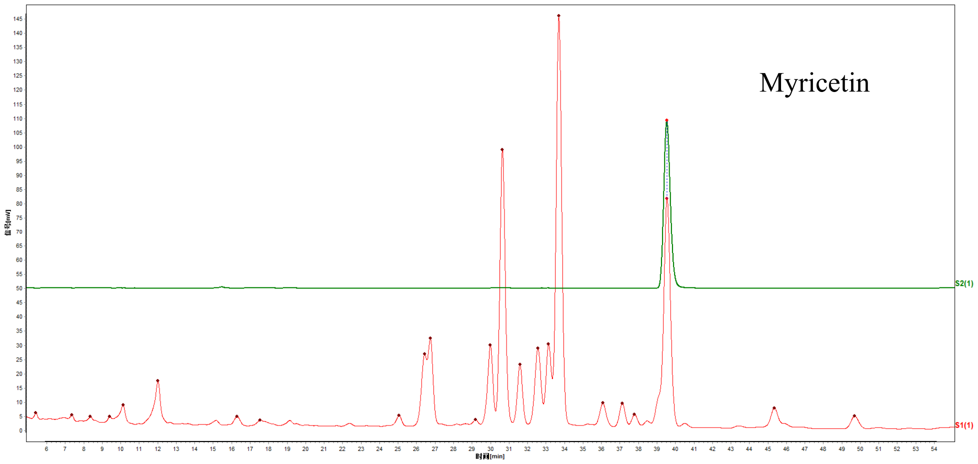
Myricetin
S2(1)
S1(1)

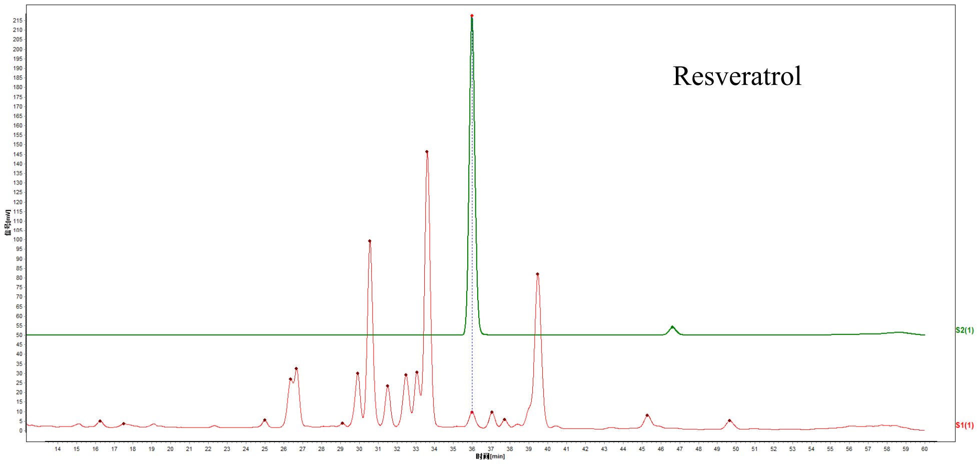
Resveratrol
S2(1)
S1(1)

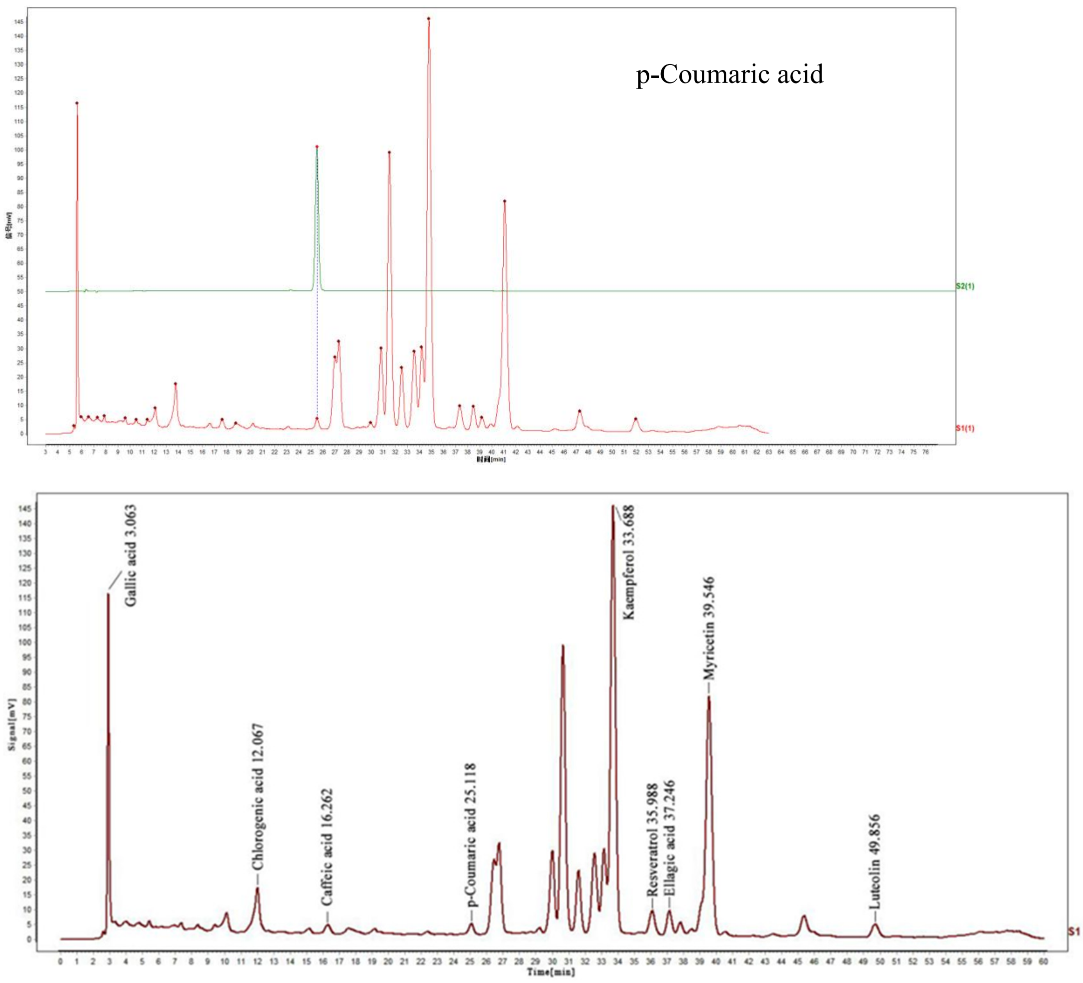

Figure S1. Composition of hazelnut leaf polyphenols versus standards
